# Nectar and oleiferous trichomes as floral attractants in *Bulbophyllum saltatorium* Lindl. (Orchidaceae)

**DOI:** 10.1007/s00709-017-1170-4

**Published:** 2017-09-24

**Authors:** Małgorzata Stpiczyńska, Bartosz J. Płachno, Kevin L. Davies

**Affiliations:** 1 Faculty of Biology, University of Warsaw, Botanic Garden Al. Ujazdowskie 4, 00-478 Warsaw, Poland; 2 Department of Plant Cytology and Embryology, Jagiellonian University in Kraków, 9 Gronostajowa St., 30-387 Kraków, Poland; 3 School of Earth and Ocean Sciences, Cardiff University, Main Building, Park Place, Cardiff, CF10 3AT UK

**Keywords:** African *Bulbophyllum*, Cell wall ingrowths, Flower morphology, Labellar micromorphology, Nectaries, Oleiferous trichomes, Section *Ptiloglossum*, Ultrastructure

## Abstract

Although many Orchidaceae have deceit flowers that produce no reward, the most common reward, when present, is nectar. *Bulbophyllum*, however, is unusual in that the labellar secretions of most species investigated to date lack sugars, and, therefore, cannot be considered true nectar. The African species *Bulbophyllum saltatorium* is an exception in that it produces not only nectar but also possesses specialized, capitate oleiferous trichomes. The nectary of *B. saltatorium* is borne on the labellum and is represented by a deep, narrow, median longitudinal groove, having a small aperture, and flanked by trichomes. Isodiametric epidermal cells lining this groove secrete nectar which collects both in the groove and on the surface of the labellum. As well as a nectary, the labellum of *B. saltatorium* also bears three types of unicellular trichomes: the longest trichomes are borne distally and abaxially; the marginal ones form a rim around the entire labellum, and finally, massive, capitate trichomes occur proximally and adaxially. These are oleiferous, containing large quantities of oil which might function as precursors of volatile components of fragrance or provide a food-reward. To the best of our knowledge, this is the first time for such oleiferous trichomes to be described for *Bulbophyllum*. Therefore, apart from their color and markings, flowers of this species are able to attract pollinators in at least two, possibly three ways: food-reward in the form of nectar; fragrance; and possibly food-rewards in the form of food-hairs.

## Introduction

Considered to be the largest orchid genus (Pridgeon et al. 2014), *Bulbophyllum* Thouars comprises some 2200 species occurring throughout tropical Africa, the Comoros, Madagascar, the Seychelles, Reunion and Mauritius, Asia, the Philippines, New Guinea, the tropical Pacific islands, Australia, New Zealand and the Neotropics, the main centers of distribution being Madagascar and New Guinea (Pridgeon et al. 2014). It is entomophilous, with morphologically diverse and intricate flowers displaying some of the most varied and complex pollination strategies to be found amongst orchids, and is pollinated by a wide range of insects, most notably, Coleoptera, Hymenoptera (mainly in Africa), such as wasps and bees, including stingless bees and ctenuchid wasps (Johansson 1974—cited in van der Cingel 2001; Dressler 1990, 1993; van der Cingel 2001; Chen and Gao 2011), but mainly Diptera, including fruit-flies, blowflies, flesh flies and signal flies or Platystomatidae (van der Cingel 2001; Tan and Nishida 2000, 2005; Tan et al. 2002; Humeau et al. 2011; Ong 2011; Ong et al. 2011 and references therein; Ong and Tan 2011, 2012; Liu et al. 2010; Stewart et al. 2014). As such, one would expect this genus to be a potentially rich source of new information about plant adaptation (Gravendeel et al. 2004; Fischer et al. 2007).

The rewardless condition is frequent amongst orchids (Jersáková et al. 2006), but when floral rewards are present, they mainly occur as nectar (Dressler 1990, 1993; Davies and Stpiczyńska 2008). True nectar (i.e. a sugar-rich, floral secretion attractive to pollinators) is uncommon in *Bulbophyllum* (personal observation). Indeed, by now, almost a century has passed since Pohl (1935) reported the occurrence of a sugar- and oil-rich secretion at the base of the labellum and adjacent flower parts of *Bulbophyllum lobbii* Lindl. (sect. *Sestochilos* (Breda) Benth. & Hook.f.) and *Bulbophyllum macranthum* Lindl. (sect. *Stenochilus* J.J.Sm.), yet since then, relatively few studies have considered food-rewards in *Bulbophyllum*. Field studies have reported food-rewards in a number of species such as *Bulbophyllum alticola* Schltr. (sect. *Hapalochilus* Schltr.), *Bulbophyllum auratum* (Lindl.) Rchb.f. (sect. *Recurvae* Benth. & Hook. f.), *B. lobbii* and *B. macranthum* (Pohl 1935; Jongejan 1994; van der Cingel 2001), but anatomical studies of the flower with ultrastructural and histochemical investigations of the secretory process are rare, in particular for African species. To date, the floral anatomy of representatives of the Neotropical sections *Didactyle* (Lindl.) Cogn., *Napellii* Rchb.f. and *Micranthae* Barb. Rodr*.* (Nunes et al. 2014, 2015, 2017) has been studied at SEM and light microscopy level, including histochemical tests, and combined histochemical, micromorphological and ultrastructural studies have been performed on members of Asian sections that include *Cirrhopetaloides* Garay, Hamer & Siegerist, *Cirrhopetalum* (Lindl.) Rchb.f. (Kowalkowska et al. 2015, 2016) and *Racemosae* Benth. & Hook.f. (Davies and Stpiczyńska 2014; Stpiczyńska and Davies 2016), and on African sections *Megaclinium* G. A. Fischer & J. J. Verm., *Oreonastes* G. A. Fischer & J. J. Verm. and *Ptiloglossum* Lindl. (Stpiczyńska et al. 2015). Of these, histochemistry revealed that lipids were abundant in the labellar cells of most species, except for those of sect. *Racemosae*, where the secretion consisted predominantly of protein-laden mucilage (Davies and Stpiczyńska 2014; Stpiczyńska and Davies 2016). In most cases, these secretions were produced by palisade-like epidermal cells lining the median, longitudinal labellar groove. Traditionally, but misleadingly (considering they had not been shown to produce a sugary liquid), these grooves have often been referred to as nectaries. One exception, however, is *Bulbophyllum schinzianum* Kraenzl. (sect. *Ptiloglossum*), whose floral secretion, on subjecting to refractometry, gave a total sugar value of 61.7% (Stpiczyńska et al. 2015), strongly indicating, given that it also produces fragrance, that it is not pseudocopulatory, as had previously been proposed (Jongejan 1994; van der Cingel 2001). Indeed, based on the form and depth of the labellar groove and the presence of fragrance and nectar, it is possible that this species is pollinated by Hymenoptera (Stpiczyńska et al. 2015), especially as these insects have been reported to visit such flowers (Johansson 1974). Conversely, Stewart et al. (2014) reported that flowers of *B. schinzianum* var. *schinzianum* are also visited by flies, and therefore, further field studies are required.

Here, we extend our investigations to a further African species, namely *Bulbophyllum saltatorium* Lindl. The hairy flowers of this species, in some respects, resemble those of *B. schinzianum*, and both are members of the same section, namely, *Ptiloglossum.* Preliminary studies, however, indicated that both species differ in the floral rewards that they produce. The aim of our present paper, therefore, is to investigate the micromorphology and histochemistry of the hirsute labellum of *B. saltatorium*, to check for the presence of floral food-rewards and to describe the tissues involved in their production.

## Material and methods

The plants of *Bulbophyllum saltatorium* used in this study were cultivated at the Botanic Garden of the Jagiellonian University in Kraków (living collections accession number O/2014/1185). Voucher material was deposited at the Herbarium of Jagiellonian University in Kraków (accession number KRA 464137).

## Microscopy investigations

In the present study, we employed microscopy techniques described in detail by Stpiczyńska and Davies (2016, and references therein). In order to determine the presence of secretory tissues and surface secretions, intact flowers were immersed in solutions of the following stains: Sudan III for lipids; ruthenium red (RR) for mucilage; and Coomassie Brilliant Blue R 250 (CBB) for proteins. The stained parts of flowers were examined by means of a Nikon SZM1000 stereoscopic microscope. Sections of secretory tissues from proximal and median parts of the labellum were subsequently examined using bright field and fluorescence light microscopy (LM). Semi-thin sections were stained using methylene blue/azure II (MB/AII) for general histology and the periodic acid-Schiff reaction (PAS) for insoluble polysaccharides. Hand-cut sections of both fresh and fixed material were stained with Sudan III, CBB, Lugol’s iodine solution (IKI), and RR for lipids, proteins, starch and mucilage, respectively. Furthermore, an epifluorescence microscope equipped with Prior 200 W lamp (Prior Scientific Instruments Ltd.) and UV-2B filter was employed for the investigation of cell walls, and in particular, the cuticle, in unstained material fixed in 70% ethanol, since observations of cells located deep in the labellar groove were difficult using SEM. The structure of the cuticle was further investigated by staining paradermal sections with auramine O (Ruzin 1999) and viewing them using blue light (FITC-Nikon cube filter).

For scanning electron microscopy (SEM), parts of the labellum were dehydrated and subjected to critical-point drying using liquid CO_2_. They were then sputter-coated with gold and examined using a Hitachi S-4700 scanning electron microscope (Hitachi, Tokyo, Japan), at an accelerating voltage of 20 kV.

Pieces of labellum from both proximal and median parts were fixed in 2.5% (*v*/*v*) glutaraldehyde/4% (*v*/*v*) formaldehyde in 0.1 M sodium cacodylate buffer (pH 7.0), washed in the same buffer and post-fixed in 1.5% (*w*/*v*) osmium tetroxide solution. They were dehydrated using a graded acetone series and embedded by means of a Spurr Embedding Kit (Sigma). Following polymerization, sections were cut at 70 nm for transmission electron microscopy (TEM) using a LKB ultramicrotome, stained with uranyl acetate and lead citrate (Reynolds 1963), and examined using a JEM 1400 (JEOL Co., Japan, 2008) transmission electron microscope, at an accelerating voltage of 80 kV.

Micrometry and photomicrography were accomplished using NIS-Elements BR software and DS-Fi1 digital camera (Nikon, Japan), and a high-resolution digital camera (CCD MORADA, SiS-Olympus, Germany) for LM and TEM images, respectively.

## Results

**Fig. 1:**
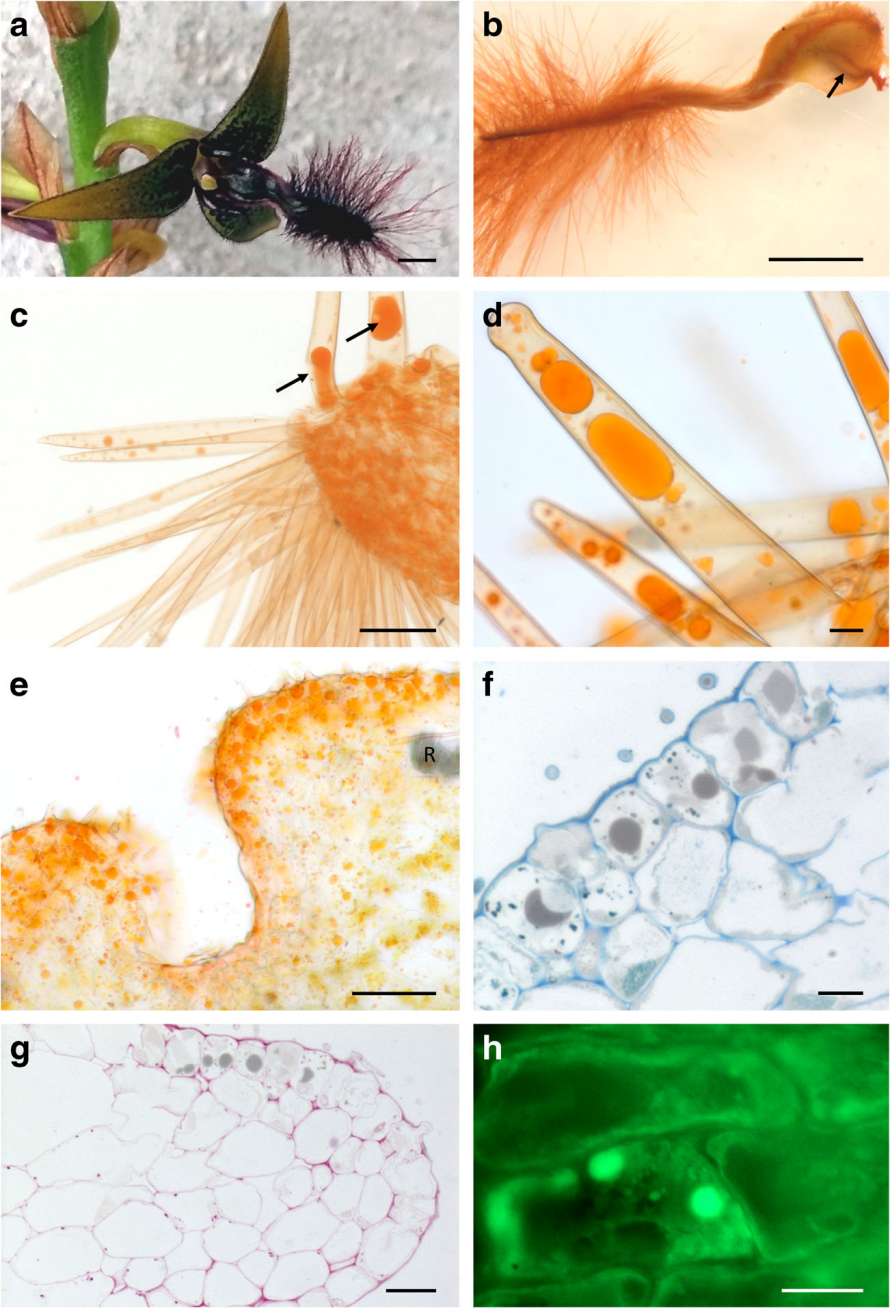
Gross floral morphology and labellar anatomy, LM. **a** Habit of flower, scale bar = 1 mm. **b** Whole labellum stained with Sudan III. The position of nectary groove (visible through the abaxial surface) is marked by an arrow, scale bar = 1 mm. **c** Short, pointed trichomes and bases of massive trichomes containing lipid droplets (arrows) following staining with Sudan III, scale bar = 50 μm. **d** Massive capitate trichomes containing lipid droplets stained with Sudan III, scale bar = 20 μm. **e** A similar preparation showing the presence of lipids in the epidermal and parenchyma cells of the proximal part of the labellum, scale bar = 50 μm. **f** Section stained with MB/AB showing large, gray lipid droplets in epidermal cells, scale bar = 10 μm. **g** Sparse, minute starch grains (PAS) and thin cellulosic cell walls occur in the lipid-secreting region. Note gray lipid droplets, scale bar = 20 μm. **h** Paradermal section stained with auramine O. Note the abundant, fluorescent green lipid droplets, scale bar = 10 μm. *R* raphides

*Bulbophyllum saltatorium* flowers are borne on a simple raceme, and each is subtended by a prominent papery bract (Fig. 1a). The bases of the sepals and petals (including the labellum) are more darkly pigmented than the remaining parts, and the flowers have a faint, sweet fragrance.

**Fig. 2:**
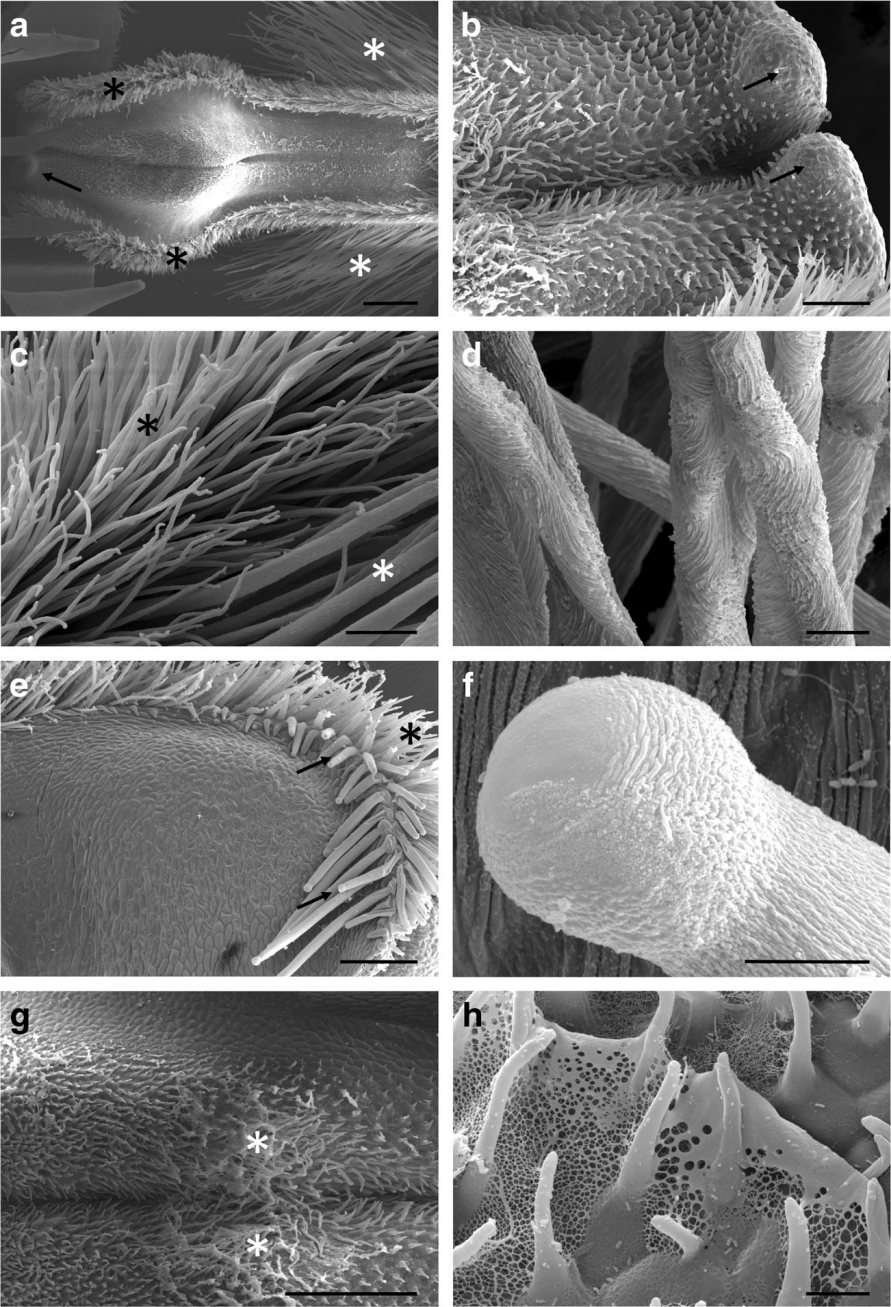
Micromorphology of the labellum, SEM. **a** Part of labellum showing median longitudinal groove and hairy callus. One of two visible basal projections marked by an arrow. Also note the border of short trichomes—black asterisks, and the long, marginal trichomes—white asterisks, scale bar = 500 μm. **b** Proximal part of labellum with two projections (arrows), scale bar = 150 μm. **c** Short trichomes (black asterisk) and smooth cuticle, and long marginal trichomes (white asterisk) of the labellum, scale bar = 60 μm. **d** Detail of the striate cuticle of long trichomes from apical part of the labellum. Note the helical arrangement of cuticular striae, scale bar = 20 μm. **e** Glabrous proximal, adaxial surface ﻿of labellum with rim of massive, capitate, oleiferous trichomes (arrows). Marginal rim of short trichomes marked by asterisk, scale bar = 200 μm. **f** Detail of swollen apex of massive, capitate hair, scale bar = 10 μm. **g** Median groove and hairy callus coated with nectar (asterisks), scale bar = 500 μm. **h** Reticulum of nectar residues between callus trichomes, scale bar = 15 μm

The labellum is densely hirsute (Fig. 1a–d). The distribution and surface features of the various kinds of labellar trichomes are shown in the SEM images (Fig. 2a–f). The longest trichomes, which measure as much as 3.5 mm in length × 18 μm in diameter, are present on the distal, abaxial surface of the labellum (Figs. 1a, b and 2a). These trichomes are unicellular with cellulosic cell walls and an irregularly or helically sculptured cuticle (Fig. 2d). In the parietal cytoplasm, small plastids occur, and the vacuoles contain anthocyanins. These trichomes do not contain any storage materials.

Marginal trichomes form a dense border or rim around the labellum. They are shorter and narrower than those described previously, measuring only some 300 μm in length × 10 μm in diameter, and have acute apices (Figs. 1c and 2a, c, e). The cuticle overlying the cellulosic cell walls is smooth and of regular thickness. Like the long trichomes, these hairs do not accumulate any substantial storage materials. Adaxially, on the proximal part of the labellum, and especially near the two proximal labellar projections, massive capitate trichomes are present, measuring 450 μm in length × 30 μm in diameter, with capitate tips (Figs. 1d and 2e, f). The cuticle overlying the trichome is irregularly striate, with horizontally orientated striae located close to the apex, which is smooth (Fig. 2f) and, as viewed using SEM, lacks pores and cracks. These trichomes contain large lipid droplets (Fig. 1c, d). Similar lipid droplets also occur in the epidermal and subepidermal parenchyma cells (Fig. 1e–h). Neither the intravacuolar contents of the three types of trichome mentioned previously, nor those of the adaxial epidermal cells displayed specific autofluorescence when exposed to UV light.

**Fig. 3:**
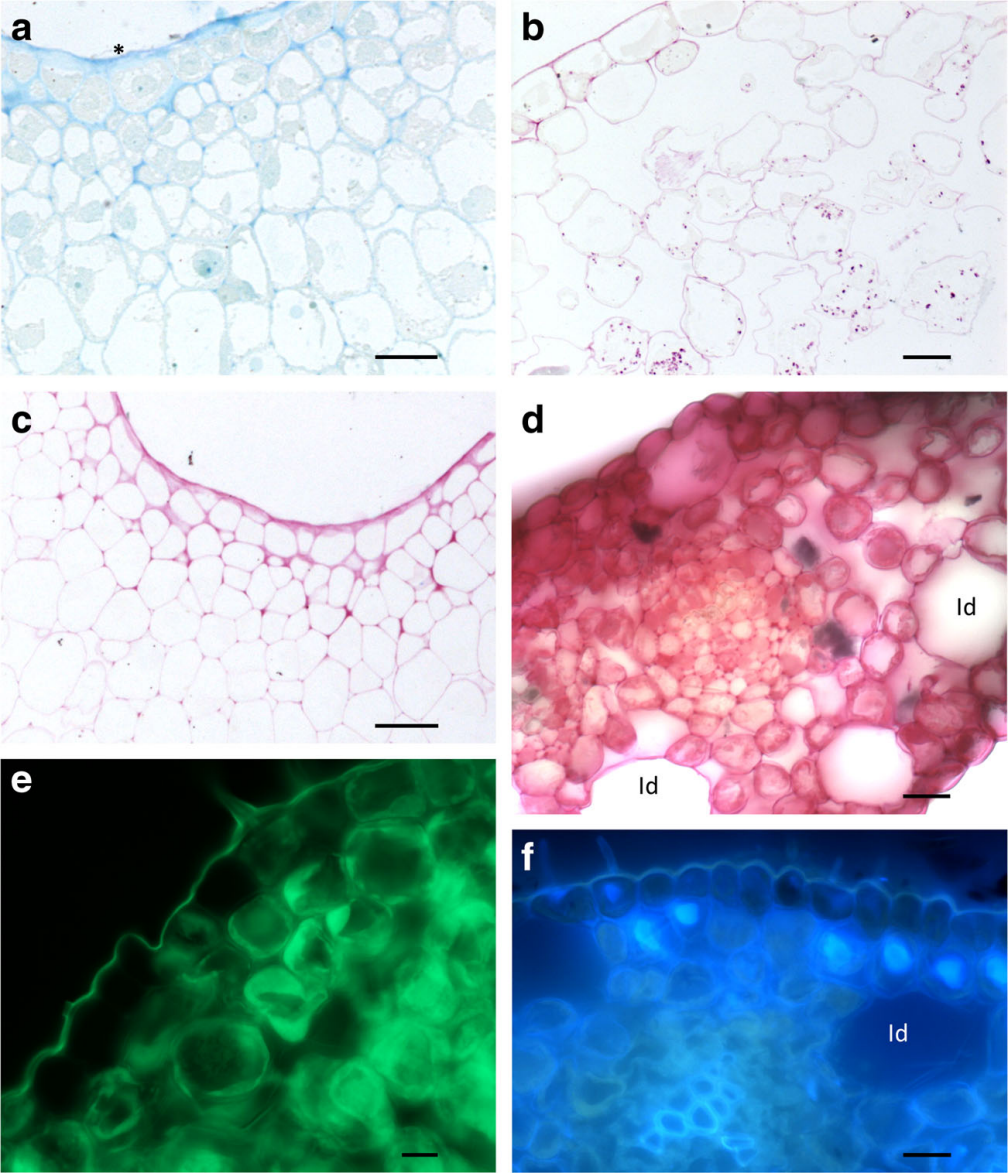
Nectar secreting region of the labellum, LM. **a** Section showing secretory epidermis lining the median groove and underlying subepidermal parenchyma (MB/AB), secretory residues marked by an asterisk, scale bar = 20 μm. **b** Epidermis and subepidermal parenchyma in the marginal part of the labellum. Starch is present in the mesophyll-like parenchyma cells (PAS), scale bar = 20 μm. **c** Section (as in **a**) stained with PAS reveals the absence of starch, but relatively thick cellulosic tangential cell walls stain with this reagent, scale bar = 20 μm. **d** Staining with RR reveals the absence of mucilage from idioblasts and the surface secretion, scale bar = 20 μm. **e** Staining with auramine O reveals an intact cuticle overlying the nectary, scale bar = 10 μm. **f** Section of callus region viewed with UV. Note autofluorescence of vacuolar contents of subepidermal cells, scale bar = 20 μm. *Id* idioblast

The median part of the labellum was coated with sweet-tasting nectar. Under SEM, residues of nectar were visible as a reticulum, or as a fine fibrous weft overlying short trichomes and conical papillae distributed alongside the median, deep, longitudinal groove, and this material was particularly abundant upon the callus (Fig. 2g, h). Marginal parts of the labellum were glabrous and lacked nectar. Transverse sections of the labellum revealed that the median, longitudinal groove of the labellum was lined with epidermal cells of mean dimensions 18.7 × 12.5 μm. These were not palisade-like, as in many other *Bulbophyllum* spp., but each contained a large nucleus and dense cytoplasm (Fig. 3a). The subepidermal parenchyma comprised several layers of compactly arranged, small isodiametric cells. Adjacent epidermal cells (located more laterally) were larger, having mean dimensions of 23.3 × 30.5 μm, and contained thin parietal cytoplasm and a large vacuole. Deeply located parenchyma cells were large, with extensive intercellular spaces, and resembled spongy mesophyll (Fig. 3b). Occasionally, large idioblasts with raphides also occur in this region. Generally, starch was lacking from epidermal cells (those lining the median groove, as well as those that are more marginally located), but was present in parenchyma (Fig. 3b, c), particularly in the vicinity of vascular bundles.

Sections of the labellum through the median longitudinal groove region tested with RR (Fig. 3d), and Sudan III, revealed that mucilage and lipids, respectively, were absent from the surface secretion. Testing with CBB also showed that the surface secretion lacked proteins. Staining sections with auramine O (Fig. 3a) and observations made using UV light (Fig. 3f) revealed an intact cuticle with no visible pores or cracks. However, cell walls stained following treatment with RR, and the dense cytoplasm of secretory cells stained with CBB.

Vacuolar contents of subepidermal parenchyma autofluoresced light blue when subjected to UV light (Fig. 3f), whereas staining with auramine O revealed the presence of minute lipid droplets (probably plastoglobuli) in epidermal and subepidermal parenchyma cells.

**Fig. 4:**
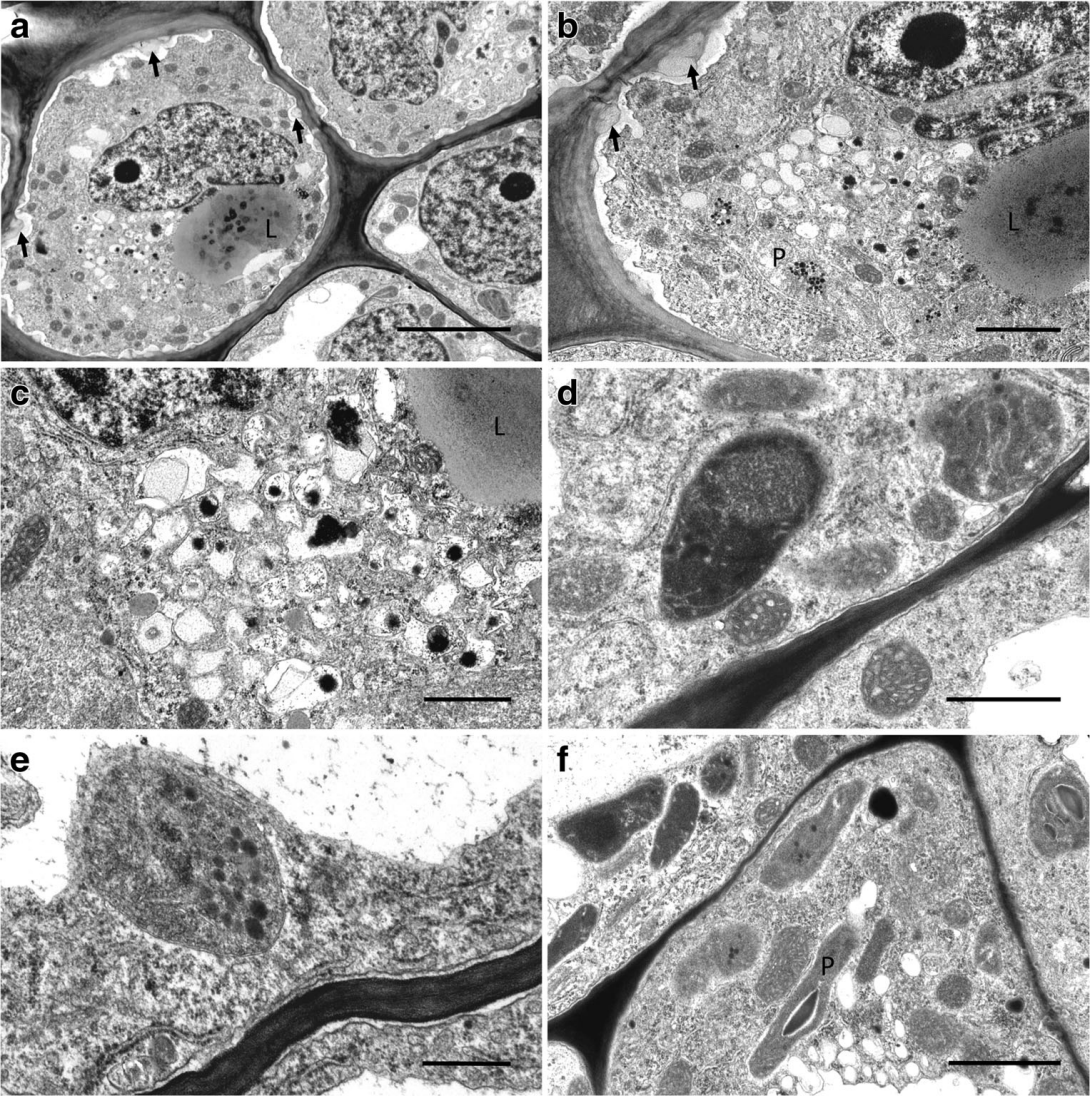
Ultrastructure of oil-secreting cells, TEM. **a** Epidermal and subepidermal parenchyma cells. Large vacuole contains lipid; the presence of cell wall ingrowths in epidermal cell marked by arrows. Mitochondria occur in the parietal cytoplasm, scale bar = 5 μm. **b** Detail of epidermal cell with large vacuole and small vesicles, both containing lipids. Numerous ER profiles occur in the cytoplasm. Cell wall ingrowths are marked by arrows, scale bar = 2 μm. **c** Detail of secretory vesicles, some of which contain electron-dense bodies, scale bar = 1 μm. **d** Starchless plastids with dense stroma and internal membranes, scale bar = 1 μm. **e** Detail of parietal cytoplasm showing RER and plastid with plastoglobuli, scale bar = 0.5 μm. **f** Subepidermal parenchyma cells with plastids enclosing few, minute starch grains, scale bar = 2 μm. *L* lipid, *P* plastid

Transmission electron microscopy of adaxial epidermal cells from the proximal part of the labellum revealed a prominent, centrally located nucleus and dense cytoplasm containing a relatively large vacuole (Fig. 4a). Lipid completely filled both this and numerous smaller vacuoles, whereas some small vacuoles and vesicles scattered throughout the cytoplasm contained electron-dense material (Fig. 4a–c). The cytoplasm contained abundant smooth (SER) and rough endoplasmic reticulum (RER), and close examination showed that both SER and RER produced small vesicles by budding. Dictyosomes were also frequently seen. The numerous mitochondria were mainly located in the parietal cytoplasm (Fig. 4a, d). Plastids were rarely observed, but when present, they contained an electron-dense stroma, few internal tubules and plastoglobuli, but starch was absent (Fig. 4b–e). By contrast, plastids in the subepidermal parenchyma contained small starch grains (Fig. 4f). Plasmodesmata connected adjoining epidermal and subepidermal cells (Fig. 4b, f). The plasmalemma was often very convoluted, resulting in the formation of an extensive periplasmic space. A distinctive feature of epidermal cells, and to a lesser degree subepidermal parenchyma cells, was the presence of cell wall ingrowths (Fig. 4a, b) distributed fairly regularly along the length of the cell walls.

**Fig. 5:**
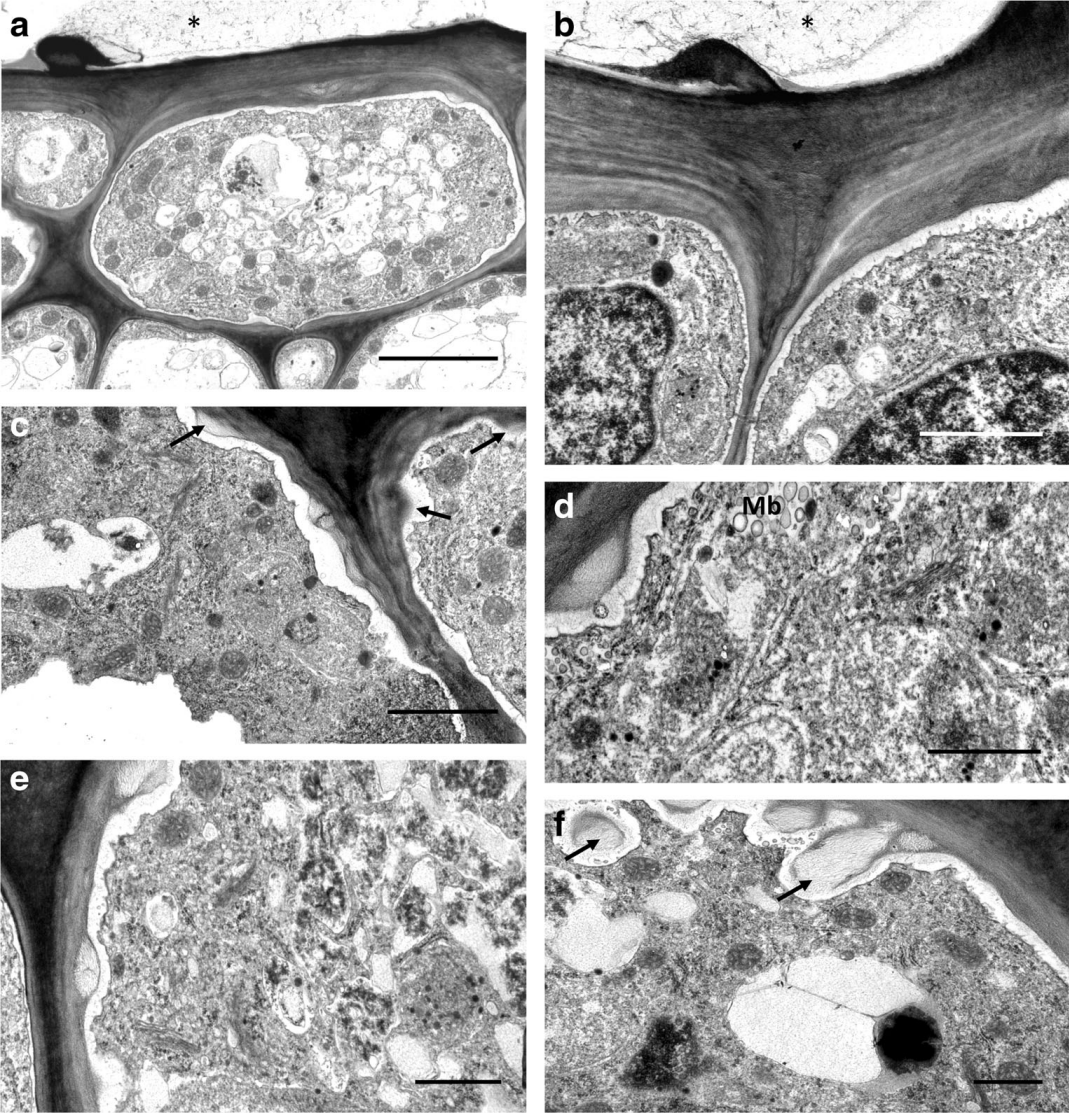
Ultrastructure of cells from nectary region, TEM. **a** Epidermal cells with thick, outer tangential wall and thin radial walls, interconnected by plasmodesmata. Surface secretion (nectar residue) marked by an asterisk, scale bar = 5 μm. **b** Detail of outer cell wall with thin cuticle and parietal cytoplasm. Note convoluted outline of plasmalemma and vesicles in periplasmic space; surface secretion marked by an asterisk, scale bar = 2 μm. **c** Parietal cytoplasm with numerous vesicles, ER and dictyosomes. Cell wall shows small ingrowths (arrows), scale bar = 2 μm. **d** Electron-dense parietal cytoplasm demonstrating the GERL system comprising dictyosomes or Golgi bodies, ER profiles and the numerous small vacuoles and secretory vesicles derived from it, scale bar = 1 μm. **e** Small vacuoles and vesicles containing osmiophilic material. Numerous dictyosomes occur in the parietal cytoplasm, scale bar = 1 μm. **f** Parietal cytoplasm with dictyosomes, ER profiles and secretory vesicles. Note cell wall ingrowths (arrows) with loose arrangement of cellulosic microfibrils, scale bar = 1 μm. *Mb* multivesicular body

These were also present in nectar-secreting cells lining the median groove (Fig. 5a–f). Nectary cells had thick, outer cellulosic walls and a thin, smooth cuticle without visible micro-channels. The surface secretion that collected on the surface of the cell wall (Fig. 5a, b) was often fibrous or weft-like, but sometimes it had a more solid appearance. As in lipid-containing cells, the plasmalemma was convoluted (Fig. 5a–f), and the periplasmic space formed contained many secretory vesicles (Fig. 5b–f). Furthermore, secretory vesicles of various sizes gathered in the parietal cytoplasm, alongside the plasmalemma, and fused with the latter (Fig. 5d–f). Abundant dictyosomes were present in the parietal cytoplasm, together with profiles of SER, RER and mitochondria, as well as small, multivesicular bodies (MVBs) (Fig. 5a). Vacuoles here were small and electron-translucent or contained flocculent material (Fig. 5a, c). They gathered predominantly in the apical parts of papillae. Occasionally, small vacuoles containing lipid were also observed. Vacuoles present in the subepidermal parenchyma were large and predominantly electron-translucent, or they contained membranous or fibrous material. Epidermal and subepidermal cells were interconnected via numerous plasmodesmata (Fig. 5a–c), the latter being particularly densely distributed on the adjoining walls of mesophyll-like parenchyma cells.

## Discussion

Of the species of *Bulbophyllum* studied to date, the median labellar groove of *B. saltatorium* most closely resembles that of *B. schinzianum* in that it is relatively deep, with a narrow aperture, and densely flanked by unicellular papillae and trichomes (Stpiczyńska et al. 2015). Since the cells lining the groove secrete a sugary liquid, like that of *B. schinzianum*, it can also be referred to as a nectary. Like the nectar of *B. schinzianum*, that of *B. saltatorium* also lacks lipid, protein and mucilage, as confirmed by histochemical tests, unlike that of Asian *B*. *lobbii*, which is reported also to contain oil (Porsch 1905; Stpiczyńska et al. unpublished).

The surface secretion of *B. saltatorium* (based on SEM and TEM observations), like that of *B. schinzianum* (based on TEM), is heterogeneous and has a fibrous component. Unlike *B. schinzianum*, however, where the groove is lined with palisade-like cells, that of *B. saltatorium* is lined with more or less isodiametric cells, indicating, perhaps, that *B. schinzianum* displays a greater degree of secretory tissue organization. Remarkably, the cellulosic cell walls of the labellum of *B. saltatorium* have pronounced wall ingrowths that are absent from those of *B. schinzianum*. Cell wall protuberances are frequently present in various types of specialized plant secretory cells (transfer cells) engaged in high rates of solute transport (Offler et al. 2002). They have also been reported for the secretory tissues of some orchid flowers, such as elaiophore cells of *Zygostates grandiflora* (Lindl.) Mansf. (subtribe Oncidiinae; *Ornithocephalus* clade—Pacek et al. 2012), the epidermal cells of petals and the labellar groove of *Bulbophyllum weberi* Ames, whose cytoplasm and surface secretion contain lipids, proteins and pectin acids/mucilage (Kowalkowska et al. 2016), as well as the nectaries of *Epipactis atropurpurea* Raf. (Pais and Figueiredo 1994). In *B. saltatorium*, cell wall ingrowths were present both in cells that accumulate lipids, and in nectar-secreting cells, indicating that they are both involved in active secretion. Transport of secretion across the plasmalemma of secretory cells with cell wall ingrowths can occur either by eccrine secretion (Płachno et al. 2017) or by granulocrine secretion, where numerous secretory vesicles congregate near to and fuse with the plasmalemma (Nepi 2007). In *B. saltatorium*, the cytoplasm of nectary cells contained abundant vesicles derived from dictyosomes and ER (GERL or Golgi–Endoplasmic Reticulum–Lysosome system), and these fused with the plasmalemma. Small vesicles were also present in the periplasmic space, and cell wall ingrowths substantially enlarge the surface area of the plasmalemma. Numerous dictyosomes and dictyosome- or Golgi-derived vesicles are also a common feature of nectary cells, and occur in orchids, such as *Platanthera* Rich. (Stpiczyńska 1997) and *Aeridinae* (Stpiczyńska et al. 2011). In contrast to nectary cells, SER profiles predominate in cells that accumulate oils, and these are frequently located close to plastids, as those that occur in other lipid-producing cells, such as those of the elaiophores of *Ornithophora radicans* (Rchb.f.) Garay & Pabst (Stpiczyńska and Davies 2008), *Z. grandiflora* (Pacek et al. 2012) and *Lockhartia* Hook. (Blanco et al. 2013).

The cell walls of the secretory cells of *B. saltatorium* (particularly the periclinal cell walls in the region of the nectary) are thick and cellulosic, resembling those reported for the nectaries of other distantly related orchid taxa, such as *Maxillaria* Ruiz & Pav. or *Hexisea* Lindl. (Stpiczyńska et al. 2004, 2005, respectively), several species of Aeridinae (Stpiczyńska et al. 2011) and *Brassavola* R. Br. (Stpiczyńska et al. 2010). Thick cellulosic cell walls in the nectary of some epiphytic orchids probably serve as a route for nectar transport onto the nectary surface. Since nectar is fundamentally an aqueous sugary solution, it may be transported within the polysaccharide matrix that constitutes the cell wall and is thus probably better protected against evaporation, especially since desiccation is a key stress factor for epiphytic plants. Therefore, the presence of thick cellulosic cell walls in unrelated epiphytic orchids is probably due to convergence. Despite this, the cuticle covering the thick outer cell walls of *B. saltatorium* is very thin, lacking the typical cuticular blisters and micro-channels seen in *B. schinzianum* (Stpiczyńska et al. 2015) and some other African and Asian species of *Bulbophyllum* investigated to date (Davies and Stpiczyńska 2014; Stpiczyńska et al. 2015; Kowalkowska et al. 2015, 2016). Therefore, by comparison, the thin cuticle of *B. saltatorium* appears to be more permeable than that of *B. schinzianum*, and this may aid nectar secretion.

Again, in both *B. saltatorium* and *B. schinzianum*, the underlying subepidermal cells are isodiametric and of similar size, and the parenchyma, which contains collateral vascular bundles and idioblasts with raphides, resembles aerial parenchyma or spongy mesophyll (Stpiczyńska et al. 2015, Stpiczynska et al. unpublished). The presence of aerial parenchyma in labella is not exclusively found in section *Ptiloglossum*, but it occurs also in other African, Asian and Neotropical *Bulbophyllum* species, and according to Teixeira et al. (2004), its presence aids movement of the labellum during pollination.

Three diverse kinds of trichome were present on the labellum of *B. saltatorium*: dense but delicate trichomes with smooth cell walls; long trichomes having irregularly sculptured cell walls, and massive capitate trichomes that accumulate large droplets of lipid. Moreover, unicellular trichomes and papillae coat the labellar callus. The first two classes of trichome, by increasing the surface area of the labellum, may also be involved in floral display and thus, the attraction of pollinators.

The massive trichomes of *B. saltatorium* are unusual in several ways. They contain large droplets of lipid, yet this material is absent from the labellar surface secretion. Therefore, it is possible that these cells are engaged in scent production since, in osmophores, the surface secretion (fragrance) tends to volatilize rapidly and thus disappear. Starch accumulation, however, which is typical of osmophore cells (Antoń et al. 2012, and references therein), was not observed here, but this is not surprising, since the starch may have been used as a source of energy for the highly metabolic process of fragrance production. Unlike the epidermal cells of many orchids that produce both fragrance and food-rewards, here, there is specialization resulting in division of labor coupled with structural modification, with trichomes producing lipid materials, whereas the cells lining the groove secrete nectar. Although the massive trichomes are laden with lipids, they differ from the elaiophore trichomes of orchids investigated so far (Pacek et al. 2012; Blanco et al. 2013) in that surface secretion is absent. To date, the production of a surface lipid-rich secretion by *Bulbophyllum* has been investigated for a number of species (Davies and Stpiczyńska 2014; Stpiczyńska and Davies 2016, and references therein; Kowalkowska et al. 2015, 2016), but oleiferous trichomes were not present in any of these taxa. Alternatively, there also remains the possibility that the oil from such trichomes, in African species, might be gathered by specialized oil-collecting bees (van der Cingel 2001), such as species of *Rediviva* (Melittidae). However, at present, there is no evidence for this in *Bulbophyllum*.

## Conclusion

In considering the species of *Bulbophyllum* investigated to date, it is possible to identify several main grades of labellar organization. These differ mainly in the type of receptacle involved in the secretion and presentation of food-rewards, ranging from a simple, shallow depression to a more highly organized, deep labellar groove that is able to secrete lipid-rich food-rewards, protein-laden mucilage or, as in *B. schinzianum* and *B. saltatorium*, nectar. Between these extremes occurs an intermediate grade comprising a shallow and wide concavity, as found in *B. lobbii* and *B. macranthum* (Stpiczyńska et al. unpublished). *Bulbophyllum lobbii*, which is fragrant, nectariferous and fly-pollinated, is considered by Christensen (1994) to have retained several features characteristic of bee-pollinated species.

The gross morphology and range of elaborate and highly specific pollination strategies is already well known for a small percentage of *Bulbophyllum* species, but with many still being discovered, and so many more remaining to be investigated fully, we can only speculate about the full diversity of the genus, not just about the floral morphology of its members, but also how they might differ at the micromorphological, anatomical, ultrastructural and biochemical levels, and the significance of each of these often slight and cryptic, but nonetheless very real differences with regard to pollinator selection and to the evolution and survival of these remarkable plants.
